# Correlation of Clinical Factors and Audiometric Characteristics with MRI Findings in Patients with Asymmetric Sensorineural Hearing Loss

**DOI:** 10.51894/001c.7005

**Published:** 2018-09-26

**Authors:** Quynh-Nhu Vu, Ellen Ko, Samuel J. Wisniewski, Garrett Carpenter, Hannah Laur, Carl Shermetaro

**Affiliations:** 1 McLaren Oakland Hospital, Pontiac, MI; 2 Statewide Campus System Michigan State University College of Osteopathic Medicine, East Lansing, MI; 3 Michigan State University College of Osteopathic Medicine, East Lansing, MI; 4 North Oakland Ear, Nose & Throat Centers P.C. and McLaren Oakland Hospital, Pontiac, MI

**Keywords:** magnetic resonance imaging, audiogram, asymmetrical sensorineural hearing loss

## Abstract

**CONTEXT:**

To identify the presence of any correlative factors between presenting symptoms and characteristics of asymmetrical sensorineural hearing loss on audiogram, and if retrocochlear pathology was identified on MRI in patients presenting in a private practice setting.

**METHODS:**

A retrospective study of patients meeting inclusion criteria who underwent MRI for asymmetric hearing loss between March 2014 to March 2017 was reviewed using Allscripts electronic health records. This data was then compiled in an excel spreadsheet and submitted for statistical analysis.

**RESULTS:**

Of the initial 687 study patients, N = 303 patients met the inclusion criteria for review. Of these 303, 48 patients (15.8%) had abnormal MRI findings. Chi-square analysis performed showed no significant association of varied clinical variables (e.g. uni and bi-lateral tinnitus, vertigo, etc.) with abnormal MRI. Point Biserial Correlation analysis revealed no statistically significant correlations, with the exception of that between AS (Left Ear) 6 kHz and MRI lesions (r = -0.115, p = 0.045). Logistic and multinomial logistic regression analysis used to calculate odds ratios showed that for patients with hearing loss at the 6 kHz (dB) level, there is a very slightly lower, statistically significant likelihood of lesions showing up on MRI (OR, 0.984 (95% CI, 0.970-0.998), p = 0.0251).

**CONCLUSIONS:**

The results lead to the conclusion that there may be an association between experiencing hearing loss at the level of 6 kHz and a slightly lower chance of the presence of retrocochlear lesion noted on MRI.

## INTRODUCTION

When a patient presents with asymmetrical sensorineural hearing loss, a variety of etiologies must be considered. These causes may arise from a number of categories including: infectious, pharmacologic ototoxicity, acoustic trauma, metabolic or autoimmune disorders, and neoplasms.^1^ The current gold standard for further evaluation of asymmetrical sensorineural hearing loss is gadolinium-enhanced magnetic resonance imaging (MRI).^2,3^ MRI is often ordered to rule out an intracranial tumor as an etiology; however, MRI is costly and otherwise has a low diagnostic yield for further evaluating asymmetric sensorineural hearing loss.^4^ Many otolaryngologists will consistently order MRI to rule out intracranial tumors out of concern for medicolegal reasons, even though a majority of these scans will return negative for a retrocochlear cause of the asymmetric hearing loss.^3^

Vestibular schwannoma, also called acoustic neuroma, is the most common tumor of the cerebellopontine angle (located between the cerebellum and pons) and often presents with unilateral or asymmetric sensorineural hearing loss (Figure 1).^5^ Other symptoms may present in association, including imbalance and tinnitus,^5^ however, these symptoms are often absent in up to 45% patients diagnosed with acoustic neuroma.^2^

**Figure:**
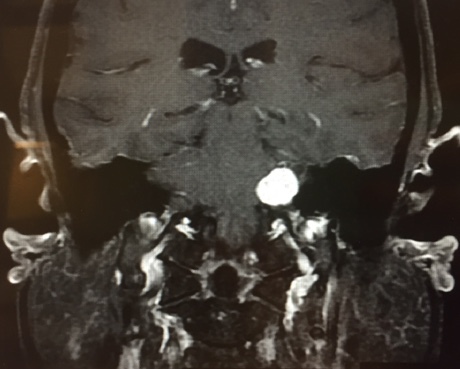
Figure 1 Acoustic Neuroma MRI brain/internal auditory canals of 19mm enhancing Ieft acoustic neuroma. Image obtained from North Oakland Ear, Nose & Throat Centers P.C.

### Purpose of Study

The goal of this study was to retrospectively identify the presence of any correlative factors between presenting symptoms and characteristics of asymmetrical sensorineural hearing loss on audiogram, and whether or not retrocochlear pathology was identified on MRI in patients presenting in a private practice setting. Findings from this research may help guide further evaluation of asymmetrical sensorineural hearing loss in the private practice setting and possibly avoid low-yield MRI testing by identifying characteristic criteria. This would, in turn, aim to decrease unnecessary health care costs.

## METHODS

After McLaren Oakland institutional review board approval was obtained, the authors (comprised of physicians and medical students) completed a query of Allscripts electronic health records from North Oakland Ear, Nose & Throat Centers. They initially identified a total of 687 patients who underwent MRI for asymmetric hearing loss between March 2014 and March 2017. A retrospective review of these patient charts was performed. Investigators were supplied with a user-specific login to access the patient charts, and data from each chart was recorded in an excel spreadsheet. Patients were assigned a unique numerical identifier on the spreadsheet to protect their identities. The specific data elements recorded from the history and physical of each chart included:

- Age
- Sex
- Onset of hearing loss (sudden or gradual)
- Presence or absence of tinnitus (unilateral or bilateral)
- Presence or absence of vertigo
- Presence or absence of disequilibrium or imbalance
- Presence or absence of aural fullness or otalgia
- Presence or absence of subjective hearing loss (unilateral or bilateral)
- Audiogram findings (hearing threshold levels for right and left ears at 250 Hz, 500 Hz, 1 kHz, 2 kHz, 3 kHz, 4 kHz, 6 kHz, 8 kHz and speech discrimination scores for right and left ears)
  - Hearing threshold levels = octave frequencies 250-8000 Hz were used^6^
  - Hz = hertz, unit of frequency
  - Speech discrimination score = reflects percentage of phonetically balanced words that a patient repeats correctly^6^
- MRI results (normal/unremarkable, incidental finding(s), or presence of vestibular schwannoma and size of lesion)

### Inclusion criteria:

1. Patients with asymmetrical sensorineural hearing loss (based on previous definitions^2,5,7,8^) on audiogram documenting at least one of the following:
  - ≥10 decibel asymmetry across three consecutive frequencies,
  - ≥15 dB asymmetry across two consecutive frequencies,
  - ≥15 dB asymmetry at 3 kHz,
  - ≥15 dB asymmetry between the average of 0.5, 1, 2, and 3 kHz, and/or
  - ≥15% difference between speech discrimination scores^7^
2. Who subsequently (after audiogram) underwent MRI to evaluate for retrocochlear causes of asymmetrical sensorineural hearing loss.

### Exclusion Criteria:

1. Patients with recent head trauma or use of chemotherapeutic or ototoxic medications.
2. Remaining patients who did not meet the inclusion criteria.

Data from these patient charts were compiled in a spreadsheet and submitted for statistical analysis to the third author (SJW) at the Michigan State University College of Osteopathic Medicine Statewide Campus System.

### Statistical Analysis:

Initial analyses assessed whether there were any correlations between the exposure (asymmetrical sensorineural hearing loss) and the outcome (retrocochlear lesion noted on MRI). Also taken into account were factors that could play a part in the relationship between the exposure and outcome. These potential confounding factors included imbalance, vertigo, tinnitus (bilateral or unilateral), the degree of hearing loss, and otalgia. As such, bivariate correlations were examined, (e.g. the hearing thresholds for the right and left ears at the various levels 250Hz, 500Hz, etc.) along with whether a normal or abnormal MRI result was observed. Logistic regression modeling was then performed using the proc logistic function, examining those correlations observed to be significant in the bivariate correlations analyses, in combination with the potential confounding variables (imbalance, vertigo, tinnitus, the degree of hearing loss, and otalgia). All analyses were performed by the third author (SJW) using SPSS Version 24. An alpha cut-off of < 0.05 was considered statistically significant for all analyses.

## RESULTS

Of the initial 687 study patients, N = 303 (44%) patients met the inclusion criteria for review. Of these 303, 48 patients (15.8%) had abnormal MRI findings (Table 1). Chi-square analysis performed showed no significant association of clinical variables with abnormal MRI (Table 2). Point Biserial Correlation analysis revealed no statistically significant correlations, with the exception of that between AS (Left Ear) 6 kHz and MRI lesions (r = -0.115, p = 0.045) (Table 3). This suggests that there may be an association between experiencing hearing loss at the level of 6 kHz and the presence of retrocochlear lesion noted on MRI. Further analyses utilizing logistic and multinomial logistic regression to calculate the odds ratio (OR) showed that for patients with hearing loss at the 6 kHz level, there was a *very slightly* lower, statistically significant likelihood of lesions showing up on MRI, OR, 0.984 (95% CI, 0.970-0.998), p value = 0.0251.

**Table:** Table 1 Descriptive Statistics

***Variable***	***Patients: N = 303***
*Age (mean)*	59 years old (SD = 13.6)
*Sex*	N = 168 (55.4%) female N = 135 (44.6%) male
*Abnormal MRI*	N = 48 (15.8%)
*Onset of symptoms*	N = 246 (81.2%) gradual N = 49 (16.2%) sudden
*Tinnitus*	N = 193 (57.1%)
	-0.7% non-specific -25.1% B/L -16.2% L -11.2% R -2.3% R > L -1.7% L > R
*Vertigo*	N = 39 (12.9%)
*Disequilibrium or Imbalance*	N = 80 (26.4%)
*Aural Fullness, Otalgia, or Plugged Feeling*	N = 118 (38.9%)
	-0.3% non-specific -7.3% B/L -17.8% L -12.2% R -0.3% R > L -0.3% L > R
*Subjective hearing loss*	N = 207 (68.3%)
	-19.1% B/L -23.8% L -15.2% R -3.0% R > L -7.3% L > R

B/L = bilateral

L = left

R = right

**Table:** Table 2 Association of Clinical Variables with Abnormal MRI*

***Variable***	***Normal MRI***	***Abnormal MRI***	***p-value***
Unilateral tinnitus	70 (23.1%)	15 (4.92%)	p = 0.591
Bilateral tinnitus	76 (25.1%)	10 (3.30%)	p = 0.206
Vertigo	30 (9.90%)	9 (2.97%)	p = 0.185
Disequilibrium or Imbalance	70 (23.1%)	10 (3.30%)	p = 0.340
Aural fullness, otalgia, or plugged feeling	103 (33.9%)	15 (4.95%)	p = 0.233

*Chi-square analyses performed

**Table:** Table 3 Association of Audiometric Variables with Abnormal MRI*

***Variable***	***p-value***
AD (Right Ear) 250	0.811
AD 500	0.580
AD 1k	0.464
AD 2k	0.411
AD 3k	0.251
AD 4k	0.230
AD 6k	0.332
AD 8k	0.279
AS (Left Ear) 250	0.879
AS 500	0.838
AS 1k	0.625
AS 2k	0.188
AS 3k	0.075
AS 4k	0.065
**AS 6k**	0.045**
AS 8k	0.079

*Point biserial correlation analyses performed

**Significant at the 0.05 alpha level

## DISCUSSION

One of the cardinal features of acoustic neuroma is the presence of an asymmetric sensorineural hearing loss, and the most frequent presenting symptoms of acoustic neuroma occurring in greater than 95% of patients is hearing loss.^9^ According to Hentschel *et al*., all patients that present with asymmetrical hearing loss or unilateral audiovestibular dysfunction will obtain an MRI, leading to a considerable amount of MRIs with negative findings as the incidence of acoustic neuroma in their screening population varies from 1% to 4.7%. This equates to more than 95% of MRIs resulting negative for acoustic neuroma.^4^

The goal of our retrospective study was to identify the presence of any correlative factors between presenting symptoms and characteristics of asymmetrical sensorineural hearing loss on audiogram, and whether or not retrocochlear pathology was identified on MRI in patients presenting in a private practice setting. Overall, results showed that there appears to be very little difference, if any, in the clinical and/or audiometric findings of those with or without retrocochlear lesions assessed via MRI in private practice setting. One correlation noted in our study was that those with hearing loss at the 6 kHz level showed a slightly lower likelihood of lesions observed on MRI. Further analysis (logistic regression) used to calculate odds ratios demonstrated a slightly lower odds that those with hearing loss at the 6 kHz level were likely to have lesions show up on MRI (p value = 0.0251; OR, 0.984; 95% CI, 0.970-0.998). However, this OR approaches very closely to 1, so much so that rounding the higher end of the confidence interval (CI) would actually be 1. This is within a margin of error and suggests that any significance observed is likely the result of statistical noise (i.e. unmeasured confounding factors).

The discussion is then what other data in the literature can help further guide evaluation of asymmetrical sensorineural hearing loss and potentially avoid uneccesary low-yield MRI testing by identifying other characteristic criteria, and thus decreasing health care costs. In reviewing the literature, Saliba et al., proposed the rule of 3,000. They state patients with asymmetrical sensorineural hearing loss of 15 dB or more at the 3 kHz warrant an investigation with MRI and that if the asymmetrical sensorineural hearing loss is less than 15 dB, a biannual audiometric follow-up is recommended.^5,8^ Although we did not find the same results in our study, the above mentioned data may provide ololaryngologists information on ways to reduce the number of negative MRIs ordered.

A limitation to this study is the limited number of our population in a private setting, as we only reviewed N = 303 patients that met our inclusion criteria. A future study with a more generalizable sample (i.e. not limited to the private setting population) could provide improved external validity.

## CONCLUSIONS

The purpose of this study was to identify possible correlations between presenting symptoms and characteristics of asymmetrical sensorineural hearing loss on audiogram, and if retrocochlear pathology was observed on MRI in patients within the setting of private practice. A retrospective chart review was conducted; following the statistical analysis of N = 303 patient charts, there was very little difference found between the clinical and/or audiometric findings in patients with or without retrocochlear lesions on MRI. This is with the exception found between those with hearing loss at the 6 kHz level and having a slightly lower likelihood of retrocochlear lesion on MRI findings. Further investigation into potential correlations between findings on clinical exam, audiogram, and MRI will be necessary. These future studies should be conducted on larger sample sizes in both private practice and non-private practice settings in hopes of creating a more concise medical decision-making process for ordering MRI to rule out retrocochlear lesion.

### Conflict of Interest

The authors declare no conflict of interest.
